# Promotion of the Toxic Action of Cyclophosphamide by Digestive Tract Luminal Ammonia in Rats

**DOI:** 10.5402/2011/450875

**Published:** 2011-07-14

**Authors:** Jury Ju. Ivnitsky, Timur V. Schäfer, Vladimir L. Rejniuk

**Affiliations:** Laboratory of Origin, Institute of Toxicology, Federal Medical Biological Agency, 1, ul. Bekhtereva, St. Petersburg, 192019, Russia

## Abstract

To estimate the influence of the digestive tract luminal ammonia pool on acute toxic effects of cyclophosphamide, the dynamics of blood ammonia, glutamine and urea level, symptoms of toxic action and the survival time have been studied in rats, intraperitoneally treated with cyclophosphamide, at the background of the gavage with non-lethal dose of ammonium acetate (12 mmol/kg, i.e., 0.35 LD_50_). Ammonium acetate enhanced the hyperammonaemic action of cyclophosphamide while promoting its lethal action: the mean survival time decreased 1.5, 2.1, 2.8, or 6.1 times at the background of cyclophosphamide i/p doses 200, 600, 1000, or 1400 mg/kg, respectively. Animals exposed to the combination of toxicants, manifested symptoms which were characteristic of the effect of lethal doses of ammonia salts. These data provide the evidence of the detrimental role of gastrointestinal luminal ammonia in the acute high-dose cyclophosphamide toxicity.

## 1. Introduction

Cyclophosphamide is nitrogen mustard-derived alkylating agent used as a cytostatic drug in the treatment of lymphomas, some forms of leukemia, and some solid tumors. Symptoms of neurotoxicity are common in myeloablative regimens of the therapy with nitrogen mustard-derived alkylating agents used as cytostatic drugs [1–4]. Some others of side effects are hepatotoxicity [5, 6] and enterotoxicity [3, 7]. The impairment of hepatic and (or) colonic barrier functions may enhance the flux of gastrointestinal ammonia into the bloodstream, thus contributing to neurotoxic effects of cytostatic drugs and restricting their endurable dose levels. Hyperammonaemia is a regular finding in shock [8, 9], and the latter is one of high-dose nitrogen mustards acute effects [10]. To elucidate the role of the digestive tract luminal ammonia in the toxic action of nitrogen mustard-derived alkylating agent cyclophosphamide, the single high-dose administration of cyclophosphamide at the background of a gavage with ammonium acetate (AA) was employed in this work.

## 2. Methods

### 2.1. Animals

Mature breedless male albino rats (4–4.5 months old, 200–240 g) from the Rappolovo breeding center of the Russian Academy of Medical Sciences were used in experiments in accordance with the regulations of performing scientific investigations on toxic action of pharmaceuticals with the use of experimental animals (by the Public Health Ministry of the Russian Federation, 1997). Within the day before the experiment rats were not fed and had the unlimited access to water. Animals were allocated randomly to experimental groups.

### 2.2. Exposure to Cyclophosphamide and Ammonium Acetate

The officinal cyclophosphamide (*Cyclophosphanum*, 200 mg per flack, AG “Biochimik”, Russia), has been dissolved in distilled water *ex tempore* and administered to rats i/p (1 mL per 100 g of body weight) in lethal doses 200, 600, 1000, or 1400 mg/kg. Using animals of the same series, it has been revealed by the authors that these doses were relevant to the mean duration of life 240, 51, 13, and 2 h, respectively. The same volume of water has been injected to control rats.

AA was administered by a single gavage (1 mL per 100 g of body weight) in nonlethal dose 12 mmol/kg (0.35 LD_50_). The same dose of sodium acetate (SA) was administered to control rats.

### 2.3. Biochemical Examinations

To assay nitrogenous intermediates, blood was deproteinized immediately by 10% trichloroacetic acid. Ammonia was determined with Nessler's reagent [11]. The glutamine concentration was calculated by the increase of ammonia content resulted from the acidic hydrolysis [12]. Urea was determined by diacetyl monoxime method using the reagent kit purchased from Olvex diagnosticum GmbH, Russia.

### 2.4. Allocation to Experimental Groups and Protocol

Two sets of experiments have been performed. Within each set of experiments, all determinations were performed within 1 day; the number of each experimental group was 6, except for the assessment of the mean survival time (11 animals per group per dose of cyclophosphamide). Each animal was subjected to a single administration of cyclophosphamide.

In set 1, the effect of cyclophosphamide on the metabolism of ammonia was estimated at the background of the increased gastrointestinal luminal ammonia pool. Ammonia, glutamine, and urea were assayed in blood obtained from the trunk by decapitation at 0.5, 1.5, or 3 h after the administration of AA and (or) cyclophosphamide (600 mg/kg). AA was administered immediately before the exposure to cyclophosphamide.

In set 2, the clinical meaning of alterations of the kinetics of gastrointestinal ammonia was elucidated by assessing the impact of gavages with AA upon clinical manifestations of toxic effects and the duration of life observed in rats after the subsequent treatment with cyclophosphamide (200, 600, 1000, or 1400 mg/kg).

### 2.5. Statistical Analysis

Differences between group mean values of metabolic indices were estimated by the two-way ANOVA (cyclophosphamide × AA) and the Fisher LSD test, that of mean survival time—by the Mann-Whitney *U* test. The data was analyzed using OriginPro 8.5 Software (Origin Lab Corporation, Northampton, Mass, USA) and presented as mean ± SE. At *P* ≤ 0.05 differences were considered to be significant.

## 3. Results

In rats, subjected to the sole gavages with ammonium or sodium salts of acetic acid (12 mmol/kg), no visible toxic effects have been noticed within subsequent 48 h. The administration of cyclophosphamide in dose 200 mg/kg had no marked effect upon animals' behavior within 18 h; 600 mg/kg resulted in the development of slowly progressing somnolence and stupor. The administration of cyclophosphamide in dose 1000 or 1400 mg/kg resulted in tremor, the loss of righting, and audiomotor reflexes within 3 h; within 1–6 h the soporose state was incidentally superposed by tonic seizures. No noticeable symptoms of intoxication were observed within 0.5 h after the administration of cyclophosphamide in any dose but 1400 mg/kg.

**Figure 1:**
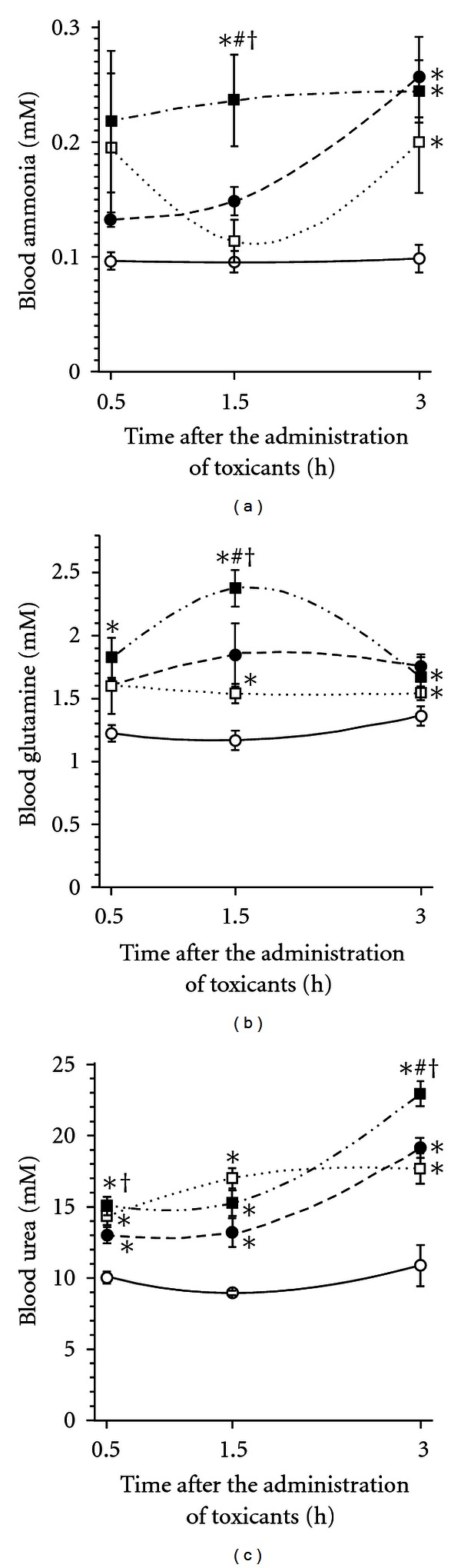
Blood ammonia, glutamine, and urea in rat after the gavage with ammonium acetate and (or) the intraperitoneal administration of cyclophosphamide, mean (SEM), *n* = 6. Opened circles: sodium acetate; closed circles: sodium acetate + cyclophosphamide; opened squares: ammonium acetate; closed squares: ammonium acetate + cyclophosphamide. Significantly different, *P* ≤ 0.05, with (*) sodium acetate group; (#) ammonium acetate group; (†) cyclophosphamide group.

The increase of blood ammonia, glutamine, and urea was observed within 3 h after the administration of cyclophosphamide and (or) AA. At the background of their combined dosing, the blood ammonia level increased 2.3 times at 1.5 h, while at the background of the separate application of cyclophosphamide this could be observed 1.5 h later. At 1.5 h after the combined toxicants' administration, the ammonia blood level has been 1.6 times higher, compared with the separate application of cyclophosphamide. When combined, AA and cyclophosphamide increased blood levels of ammonia, glutamine (at 1.5 h), and urea (at 3.0 h) more markedly than that when separately applied (Figure 1).

**Figure 2:**
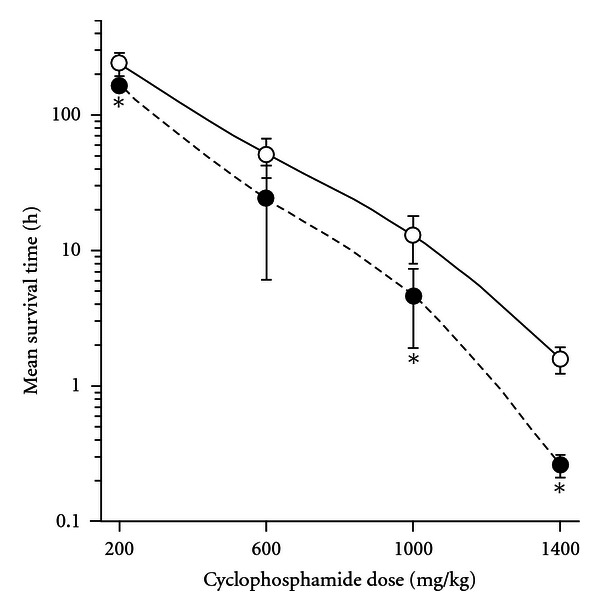
The mean survival time of rats after the gavage with ammonium acetate and (or) the intraperitoneal administration of cyclophosphamide, mean (SEM), *n* = 11. Open circles: sodium acetate + cyclophosphamide (the control group); closed circles: ammonium acetate + cyclophosphamide. Significantly different with the control group: (*) *P* ≤ 0.05.

The pretreatment with nonlethal dose of AA enhanced the lethal action of cyclophosphamide: the mean survival time of rats, which obtained cyclophosphamide in doses 200, 600, 1000, or 1400 mg/kg, decreased 1.5, 2.1, 2.8, or 6.1 times, respectively (Figure 2). Animals, subjected to the combined dosing, manifested symptoms which were characteristic of the effect of lethal doses of ammonia salts, such as exophthalmos, transient excitation, trembling, replaced by opistotonus and apnea, despite the fact that no marked symptoms of intoxication were observed in intact rats which obtained the same dose of AA.

## 4. Discussion

The digestive tract is a primary ammonia pool in the human body. Though depending on the constitution of the indigenous gut microflora, the luminal ammonia production varies largely among individuals [13, 14]. On an average, 4 g of ammonia comes from the gut into a portal blood and eventually are absorbed by the liver during a 24-h period [15]. 

Normally, luminal ammonia is absorbed readily by gastric [16], iliac [17], and colonic [18] mucosa. The hepatic vein blood ammonia level depends linearly on a portal ammonia level [19], and the latter on a luminal ammonia concentration [16]. The caval blood ammonia level may have the similar dependence because of the substantial rate of the transperitoneal ammonia translocation [20]. Hence, the flux of gastrointestinal ammonia into the common bloodstream and then into the brain may vary depending on the digestive tract luminal ammonia pool. This must be of a special importance at the background of the impairment of the intestinal mucosa barrier function, which is characteristic of the systemic action of both nitrogen and sulfur mustards [3, 7, 21].

Our study showed that the pronounced hyperammonaemic effect could be seen at 3.0 h after the administration of cyclophosphamide. When the latter was combined with AA, the ammonia level exceeded that in case of the separate application of cyclophosphamide (Figure 1). In intact rat, the luminal ammonia pool of the digestive tract amounts to 1.1 mmol/kg (calculated by the data of [22]). Hence, the gavage with AA was sufficient to increase it by an order. This indicates the positive correlation between the hyperammonaemic action of cyclophosphamide and the luminal ammonia pool of the digestive tract.

In rats, the inhalation of methyl-bis (*β*-chloroethyl) amine has been reported to inhibit the liver urea synthesis [23]. Hypothetically, the similar effect could contribute to hyperammonaemia observed in this work, Though cyclophosphamide increased not only the blood level of ammonia but that of glutamine and urea as well (Figure 1). So, the present data provide no evidence of the failure of glutamine and/or urea synthesis as a major contributive cause of observed hyperammonaemia. Therefore, the latter may be attributed, mainly, to the impairment of the gastrointestinal barrier function.

The enhancement by gavages with AA of the hyperammonaemic action of cyclophosphamide promoted its lethal action; the degree of the promotion correlated positively with the dose of cyclophosphamide (Figure 2). The involvement of ammonia in the lethal effect was clearly indicated by the accentuation of symptoms which were the characteristic effect of lethal doses of ammonia salts. The maximal decrease of the mean survival time has been demonstrated at the background of cyclophosphamide-induced severe neurological disorders. Accordingly, the maximal detrimental effect of the redistribution of gastrointestinal luminal ammonia should be expected in heavy exposure to mustard gas, bringing to the development of acute neurological disorders. Such cases have been observed in Iran-Iraqi war [24] as well as in Bari Harbor accident on December 2nd, 1943, where first deaths occurred 18 h after the exposure [25].

Ammonia gas is very aggressive and cytotoxic until it is converted into ammonium ions, which cells can tolerate at millimolar levels. Ammonia has a detrimental effect on the Krebs cycle [26]. The ensuing restriction of the cellular energy supply may be potentiated by the draining of the cellular NAD^+^ pool by the alkylating agent-mediated activation of poly-ADP-ribosylation in various cells [27]. Hence, the digestive tract luminal ammonia pool might be involved in the development of not only acute neurological disorders but also some other systemic effects. This may explain the shortening by gavages with AA of the mean survival time in rats treated with cyclophosphamide in dose which was not apparently neurotoxic (200 mg/kg).

Therefore, in rat, the size of the luminal ammonia pool of the digestive tract constitutes one of leading factors contributing to the severe toxicity of cyclophosphamide.

## 5. Conclusions

In rats, the increase of the digestive tract luminal ammonia pool by the mean of an oral gavage with nonlethal dose of ammonium acetate enhanced the hyperammonaemic action of cyclophosphamide while promoting its lethal action. Animals, exposed to the combination of toxicants, manifested symptoms which were characteristic of the effect of lethal doses of ammonia salts. These data provide the evidence of the detrimental role of gastrointestinal luminal ammonia in the acute high-dose cyclophosphamide toxicity. So, before addressing the challenge of cyclophosphamide myeloablative therapeutic regimens, it is important to consider the possibility of the promotion of the toxic action of cyclophosphamide by digestive tract luminal ammonia.
